# Gemcitabine plus nab-paclitaxel for advanced pancreatic cancer after first-line FOLFIRINOX: single institution retrospective review of efficacy and toxicity

**DOI:** 10.1186/s40164-015-0025-y

**Published:** 2015-10-07

**Authors:** Yue Zhang, Howard Hochster, Stacey Stein, Jill Lacy

**Affiliations:** Division of Hematology/Oncology, Stony Brook University School of Medicine, HSC T15, RM 040, Stony Brook, NY 11794-8151 USA; Smilow Cancer Center, Yale University School of Medicine, New Haven, CT 06519 USA

**Keywords:** Pancreatic cancer, Second line treatment, Gemcitabine plus nab-paclitaxel, FOLFIRINOX

## Abstract

**Background:**

We conducted a retrospective review of the dose, toxicity, and efficacy of second line gemcitabine plus nab-paclitaxel (G + Nab-P) after FOLFIRINOX in patients with metastatic and locally advanced unresectable pancreatic cancer.

**Methods:**

In this retrospective study, we included all patients with locally advanced unresectable or metastatic pancreatic cancer who were treated at Yale Cancer Center with G + Nab-P between 12/2011 and 12/2013 after receiving first line FOLFIRINOX. For each patient, demographics, prior therapy, doses of G + Nab-P (cumulative doses and dose intensity relative to full dose G + Nab-P), hematologic toxicities, best response by RECIST, time to treatment failure (TTF), and survival were compiled. Median TTF and overall survival (OS) were calculated by Kaplan–Meier method.

**Results:**

28 patients were treated with G + Nab-P after first line FOLFIRINOX. The median TTF was 12.0 weeks (range 2.0–36.0), and the median OS was 23.0 weeks (range 2.1–85.4). Five patients had a partial response (response rate 17.9 %), and 28.6 % of patients had stable disease for ≥7 weeks. A decline in CA 19-9 and CEA by >30 % was observed in 13 (46.4 %) and 11 (39.3 %) patients, respectively. The median relative dose intensities were 62.4 and 57.5 % for G and Nab-P, respectively. Grade ≥3 hematologic toxicities included neutropenia in 17.9 %, anemia in 25.0 %, and thrombocytopenia in 25.0 % of patients.

**Conclusions:**

Second line G + Nab-P following FOLFIRINOX is feasible, and demonstrated modest activity and clinical benefit in advanced pancreatic cancer. The optimum sequencing and dosing of these active regimens warrants further evaluation in prospective trials.

## Background

For decades the prognosis of advanced pancreatic cancer has been dismal with 5 year survival rates of approximately 2 and 4 % for metastatic and locally advanced unresectable pancreatic cancer, respectively [1]. Gemcitabine had been the standard of care for metastatic pancreatic cancer since 1997 based on modest survival benefit compared to bolus 5-fluorouracil (5.6 vs. 4.4 months, P = 0.002) and clinical benefit response (23.8 vs. 4.8 %, P = 0.002) [2]. In 2010 a new standard of care emerged when the combination regimen FOLFIRINOX (oxaliplatin, irinotecan, fluorouracil, and leucovorin) was shown to significantly improve the survival of fit patients with metastatic pancreatic cancer compared to gemcitabine (11.1 vs 6.8 months, HR = 0.57, P < 0.001) [3]. More recently the two drug combination of gemcitabine with nab-paclitaxel (G + Nab-P) was also shown to increase survival compared to gemcitabine alone in previously untreated patients with metastatic pancreatic cancer (8.5 vs 6.7 months, HR = 0.72, P < 0.001) [4].

Despite the increasing use of FOLFIRINOX for advanced pancreatic cancer since 2010, there are no published studies of efficacy or tolerability of second line regimens in patients who had received first line FOLFIRINOX. The use of gemcitabine with G + Nab-P is an attractive option in FOLFIRINOX-treated patients, given its activity in previously untreated patients with metastatic pancreatic cancer [3]. However, the optimum dose, tolerability, and efficacy of second-line G + Nab-P after first line FOLFIRINOX are unknown. In this single institution retrospective review, we evaluated the tolerability and efficacy of second line G + Nab-P in patients with metastatic or locally advanced unresectable pancreatic cancer who had received first line FOLFIRINOX.

## Methods

We conducted a retrospective review of all patients with locally advanced unresectable or metastatic pancreatic cancer who were treated at Yale’s Smilow Cancer Center between December 2011 and December 2013 with second line G + Nab-P after previous therapy with FOLFIRINOX. This study was approved by Yale IRB. The data for each patient was obtained by review of the patients’ electronic medical records and cross-sectional imaging studies. For each patient, we assessed their demographics, performance status, prior therapy, doses of G and Nab-P, the relative dose intensities of G and Nab-P (the proportion of the administered cumulative dose relative to the planned cumulative dose of G 1000 mg/m^2^ and Nab-P 125 mg/m^2^ days 1, 8, and 15, every 4 weeks), hematologic toxicities, best response by RECIST (version 1.1), time to treatment failure (TTF), and overall survival (OS). All response assessment scans were reviewed by the investigators with independent on-site radiologists. TTF was defined as time from first treatment with G + Nab-P to disease progression, unacceptable treatment toxicity (per treating physician’s assessment), patient preference, or death. OS was defined as time from first treatment with G + Nab-P to death. The median TTF and OS were determined using Kaplan–Meier method. We also determined the overall response rate and the median relative dose intensities of G and Nab-P.

## Results

### Patient characteristics

Between December 2011 and December 2013, 28 patients with advanced pancreatic adenocarcinoma were treated at the Yale Smilow Cancer Center with second line G + Nab-P after receiving first line FOLFIRINOX. The demographics and disease characteristics at the initiation of second line G + Nab-P are shown in Table 1. The median age was 61 years (range 50–74). Five patients (17.9 %) had locally advanced disease and 23 (82.1 %) had metastatic disease. Eleven patients (39.2 %) had metastasis in liver, three (10.7 %) in lung, five (17.9 %) in peritoneum, one (3.6 %) in duodenum, one (3.6 %) in liver and peritoneum, one (3.6 %) in liver/lymph nodes/bone, one (3.6 %) in lung and peritoneum. Twenty-seven patients had an Eastern Cooperative Oncology Group (ECOG) performance status of 0 or 1. Ten patients had received prior radiation therapy (50.4 Gy), and five patients had prior surgical resection of the primary tumor. In addition to FOLFIRINOX, prior chemotherapy included gemcitabine in the adjuvant setting in two patients, gemcitabine as a radiosensitizing agent in two patients, and capecitabine as a radiosensitizing agent in eight patients. Twenty-seven patients had radiographic evidence of disease progression when G + Nab-P was initiated; one patient with locally advanced disease began G + Nab-P with stable disease after chemoradiation. The median number of prior FOLFIRINOX cycles was 12 (range 5–46). The best response to FOLFIRINOX included eight patients with a partial response (PR), eighteen patients with stable disease (SD), one patient with progressive disease (PD) and one patient who was inevaluable. The median interval between the last cycle of FOLFIRINOX and initiation of G + Nab-P was 5.4 weeks in the entire cohort (range 1.7–40.3) and 17.4 weeks in the locally advanced cohort (range 5.0–40.3). Eighteen patients started second line G + Nab-P within 3 months of disease progression on FOLFIRINOX. In ten patients, G + Nab-P was initiated more than 3 months after progression of disease on FOLFIRINOX, including eight patients who had received chemoradiation after FOLFIRINOX and two patients who had received peri-operative FOLFIRINOX. Patient who received chemoradiation started G + Nab-P at least 2 months after completion of chemoradiation.

**Table 1:** Patient characteristics at initiation of gemcitabine and nab-paclitaxel

Age, years	61 (50–74)
Sex, no (%)	
Male	11 (39.3 %)
Female	17 (60.7 %)
Disease, no (%)	
Metastatic	23 (82.1 %)
Locally advanced	5 (17.9 %)
ECOG performance status, no (%)	
≤1	27 (96.4 %)
≥2	1 (3.6 %)
Median no of FOLFIRINOX cycles (range)	12 (5–46)
Median interval from last FOLFRINOX to initiation of G + Nab-P, weeks (range)	5.4 (1.7–40.3)
N = 28	

### Dosing and drug delivery

Initial dosing and subsequent dose modifications were at the discretion of the treating physician. Patients received a median of seven doses of G (range 1–27) and six doses of Nab-P (range 1–21). The initial doses of G and Nab-P were reduced compared to the published full doses in 13 (46.4 %) and 28 (100 %) patients, respectively. Nab-P was started at ≤100 mg/m^2^ in all patients. Fifteen and ten patients required subsequent dose reductions of G and Nab-P, respectively. No patients received escalating doses of G or Nab-P after initial dosing or subsequent dose reductions. Doses were reduced for hematologic toxicities (G in 15 patients and Nab-P in 10 patients), fatigue (two patients), and neuropathy (one patient). The median relative dose intensities of G and Nab-P were 62.4 and 57.5 %, respectively (Table 2).

**Table 2:** Dose reductions and dose intensity

Chemotherapy	Dose reduction at initiation, no (%)	Dose reduction after initiation, no (%)	Median relative dose intensity (%)^a^
Nab-paclitaxel	28 (100)	10 (35.7)	57.5
Gemcitabine	13 (46.4)	15 (53.6)	62.4

^a^Relative dose intensity is the proportion of the administered cumulative dose relative to the planned cumulative dose of G 1000 mg/m^2^ and Nab-P 125 mg/m^2^ days 1, 8, and 15 every 4 weeks

### Efficacy

Nineteen patients have died, four patients have discontinued G + Nab-P, and five patients were still receiving G + Nab-P at the time of the analysis. The median TTF was 12.0 weeks (range 2.0–36.0). The median OS was 23.0 weeks (range 2.1–85.4). The median time to the first response assessment scan was 8.9 weeks (range 4.4–15), and subsequent scans were obtained every 2 months. There were no complete responses. Five patients (17.9 %) achieved a partial response, and eight patients (28.6 %) had stable disease for ≥7 weeks. In the five patients with locally advanced disease, two patients had stable disease, one patient had progressive disease, and two patients were inevaluable for response. In the 23 patients with metastatic disease, five patients achieved a partial response, six patients had stable disease, eleven patients had progressive disease, and one patient was inevaluable. Thirteen (46.4 %) and eleven (39.3 %) patients had >30 % decrease in CA 19-9 and CEA, respectively (Table 3). Reasons for treatment discontinuation were disease progression (n = 18), patient preference (n = 2), and declining performance status (n = 3).

**Table 3:** Efficacy of second line gemcitabine and nab-paclitaxel

Median time to treatment failure, weeks	12.0 (2.0–36.0)
Median overall survival, weeks	23.0 (2.1–85.4)
Response by RECIST, n (%)	
Partial response	5 (17.9)
Stable disease	8 (28.6)
Progressive disease	12 (42.8)
Inevaluable	3 (10.7)
Serologic response, n (%)^a^	
CA 19-9	13 (46.4)
CEA	11 (39.3)

^a^>30 % decrease from pre-treatment baseline

### Safety

Patients were evaluated for toxicities at each treatment with history, physical examination, performance status, complete blood count, and metabolic panel. Treatment was discontinued at the discretion of the treating physician for unacceptable toxicity, progression of disease, or patient preference. There were no treatment-related deaths. Grade ≥3 hematologic toxicities included neutropenia 17.9 %, anemia 25.0 %, and thrombocytopenia 25.0 %. Eleven patients (39.3 %) received granulocyte colony-stimulating factor (G-CSF) support during their treatment (Table 4). There were no episodes of neutropenic fever. One patient discontinued Nab-P due to worsening neuropathy. Two patients required dose attenuations for fatigue.

**Table 4:** Grade 3 and 4 hematologic adverse events

Neutropenia, G-CSF support	5 (17.9 %), 11 (39.3 %)
Anemia	7 (25.0 %)
Thrombocytopenia	7 (25.0 %)

Data are expressed as the number (percentage of total group)

## Discussion

FOLFIRINOX and G + Nab-P have been shown to be more effective than gemcitabine monotherapy as first-line therapy in patients with previously untreated metastatic pancreatic cancer, extending survival to 11.1 and 8.5 months, respectively, compared to approximately 6.7 months with gemcitabine [2–4]. Despite the increasing and widespread use of FOLFIRINOX in fit patients with advanced pancreatic cancer, there is a paucity of data regarding second line regimens after first line FOLFIRINOX. The use of the FOLFIRINOX and G + Nab-P regimens in sequence is an attractive option to maximize disease control and survival.

In this single institution retrospective review, second line G + Nab-P demonstrated modest activity in patients with advanced pancreatic cancer who had previously been treated with FOLFIRINOX. The response rate of 17.9 % exceeds the response rate of single agent gemcitabine in the first line setting [2], and 46 % of patients achieved disease control (PR + SD) for at least 7 weeks. The TTF and OS of 2.8 and 5.2 months, respectively, are similar to the benefit seen with gemcitabine in the first line setting.

These results represent the largest reported experience with G + Nab-P after FOLFIRINOX in the literature. There are two published case reports of the use of G + Nab-P after FOLFIRINOX [5, 6]. In addition, two smaller retrospective series with 12 and 7 patients treated with second line G + Nab-P after prior FOLFIRINOX have been reported [7, 8].

Given the toxicities of FOLFIRINOX, concerns have been raised that patients will not tolerate a second line regimen, particularly, a doublet regimen. The majority of the patients in this study had received FOLFIRINOX for at least 6 months and were still able to tolerate this second line doublet. Notably, all patients in this study started at a reduced dose of Nab-P (≤100 mg per square meter), and thirteen patients (46.4 %) started G at a reduced dose as well. Ten and fifteen patients required subsequent dose reductions of Nab-P and G, respectively, primarily for hematologic toxicities, and 39.3 % of patients required growth factor support. The median dose intensities of G and Nab-P, 62.4 and 57.5 %, were substantially lower than reported for G and Nab-P in previously untreated patients (75 % for G and 81 % for Nab-P) [4]. Despite substantial myelosuppression, we observed no episodes of neutropenic fever or life-threatening anemia or thrombocytopenia. Although the grade of neuropathy was not documented in all patients at initiation of G + Nab-P, only one patient discontinued Nab-P due to worsening neuropathy, and this patient had neuropathy at initiation of G + Nab-P. Two patients required dose attenuations because of fatigue.

There has been no established standard of care for second line therapy in advanced pancreatic cancer. Multiple prospective trials examined a number of single agents (5-FU, irinotecan, taxanes, erlotinib, platinum agents) and doublet combinations after disease progression on gemcitabine [9–16]. A meta-analysis of 34 studies published between 2000 and 2012 confirmed modest benefit of second line chemotherapy compared to best supportive care (OS 6 vs. 2.8 months, P = 0.01) [17]. A randomized phase II study of modified FOLFIRI and modified FOLFOX after gemcitabine showed favorable efficacy and toxicity profiles with median PFS and OS of 8.3 and 16.6 weeks, respectively, in the FOLFIRI arm, and 6.0 and 14.9 weeks, respectively, in the FOLFOX arm [18]. Thus, the efficacy of G + Nab-P after FOLFIRINOX in our study is similar to what has been reported for second line therapy after less effective first line gemcitabine. This observation suggests that initial treatment with the multi-drug FOLFIRINOX regimen does not limit the use or compromise the efficacy of second line chemotherapy compared to initial treatment with gemcitabine.

Although the efficacy of second line G + Nab-P in our study is similar to what has been reported for second line therapy after gemcitabine, it is substantially lower than the efficacy of G + Nab-P in previously untreated patients (TTF 2.8 v 5.5 months; OS 5.3 v 8.5 months) [4]. The reductions in dose intensity of both G and Nab-P, greater tumor burden, and increased likelihood of chemotherapy resistance in the second line setting, all may have adversely impacted efficacy compared to G + Nab-P in untreated patients.

The limitations of this study include its retrospective design, the small number of patients identified for analysis, the heterogeneity of the patients with inclusion of locally advanced and metastatic disease, and the lack of consistent dosing or dose modifications. The choice of a second line regimen was at the discretion of the treating physicians; the majority of FOLFIRINOX-treated patients received non-Nab-P containing second line gemcitabine-based regimens during the time period of this analysis, prior to the approval of Nab-P by the Food and Drug Administration for pancreatic cancer. However, all patients who were treated at this academic center between 12/2011 and 12/2013 with second line G + Nab-P after first line FOLFIRINOX were included in this analysis, without any selection bias.

## Conclusions

At present, we do not have any validated predictive biomarkers to guide the selection of initial chemotherapy (FOLFIRINOX or G + Nab-P) for patients with advanced pancreatic cancer. Thus, it will be important to explore the optimum sequencing of these regimens, as well as the appropriate dosing, tolerability, and efficacy in prospective trials. The efficacy of G + Nab-P after first line FOLFIRINOX in this retrospective review is encouraging. Our findings demonstrate the feasibility of a sequential strategy of FOLFIRINOX followed by G + Nab-P and support further evaluation of this sequence in a prospective trial to establish the optimum dosing, tolerability, and efficacy.
